# ChildLens: An egocentric video dataset for activity analysis in children

**DOI:** 10.3758/s13428-026-02982-6

**Published:** 2026-04-13

**Authors:** Nele-Pauline Suffo, Pierre-Etienne Martin, Anas Suffo, Daniel Haun, Manuel Bohn

**Affiliations:** 1 Institute of Psychology in Education, Leuphana University Lüneburg, Universitätsallee 1, 21335 Lüneburg, Germany; 2 Comparative Cultural Psychology, Max Planck Institute for Evolutionary Anthropology, Deutscher Platz 6, 04103 Leipzig, Germany

**Keywords:** Child development, Naturalistic observation, Egocentric video-audio dataset, Multimodal learning

## Abstract

We present ChildLens, an egocentric video and audio dataset with detailed annotations for activities of naturalistic everyday experiences in children aged 3 to 5 years. A total of 109 h were recorded from 62 children in their home environment using a 140° wide-lens camera equipped with a microphone integrated in a child-friendly vest. Annotations include five location classes and 14 activity classes, covering audio-only, video-only, and multimodal activities. Good benchmark performance of two state-of-the-art models on the dataset—the Boundary-Matching Network for temporal activity localization and the Voice Type Classifier for detecting and classifying speech in audio—speak to the quality of the annotations. The ChildLens dataset will be freely available for research purposes via an institutional repository. It provides rich data to advance computer vision and audio analysis techniques and thereby removes a critical obstacle to studying the everyday context of child development, listed on the ChildLens website: https://www.eva.mpg.de/comparative-cultural-psychology/technical-development/childlens/.

## Introduction

In developmental psychology, everyday experiences play a central role when theorizing about the causes and dynamics of developmental change (Carpendale & Lewis, 2020; Heyes, 2018; Piaget, 1964; Rogoff et al., 2018; Smith et al., 2018; Tomasello, 2009; Vygotsky, 1978). Famously, the two central processes in Piaget’s theory of cognitive development—assimilation and accommodation—describe how children’s cognitive abilities change in line with the experiences they make (Piaget, 1964). Vygotsky emphasized the role of social interactions between children and (knowledgeable) adults for the acquisition of culturally relevant knowledge (Vygotsky, 1978). Contemporary theorists rest on similar ideas. For example, Tomasello (2009) pointed out how everyday social interactions, particularly those involving shared intentionality, foster uniquely human forms of communication, cooperation, and cognition. For Heyes (2018), culturally evolved “cognitive gadgets” are transmitted via language in everyday conversations between adults and children. In this paper, we present a new dataset that facilitates the development of automated techniques to scale up the study of these naturalistic everyday experiences.

To illustrate the link between everyday experience and development, we will use a few examples from the domain of language development. Across languages and cultural settings, the amount of language children hear is related to how much language they produce (Bergelson et al., 2023). There is also a relationship between the amount of conversational turn-taking children are engaged in and their vocabulary growth (Donnelly & Kidd, 2021; Ramírez et al., 2020). Roy et al. (2015) showed that words that are heard in distinct contexts at distinct times are more likely to be learned. Ruffman et al. (2023) used head-mounted video cameras to study how repeated behaviors in everyday life correlate with the acquisition of mental state vocabulary. Taken together, such studies illustrate how important it is to study naturalistic everyday experiences to understand children’s development. However, studies linking everyday experience and development are still vastly underrepresented (De Barbaro & Fausey, 2022; Rogoff et al., 2018), as they come with a set of unique challenges.

Perhaps the most significant obstacle in this field is the extensive amount of data needed to comprehensively study children’s everyday experiences. Traditional methods, such as manual annotation, are time-consuming and impractical for large-scale datasets. To address this, computer vision or natural language processing (NLP) models offer scalable solutions for analyzing social interactions and behaviors. For instance, OpenPose (Cao et al., 2021) allows the tracking of human body, face, and hand poses, which provides valuable insights into gestures and engagement. YOLOv8 (Redmon et al., 2016) offers efficient object detection, and models like I3D (Carreira & Zisserman, 2017) provide an automated solution for classifying activities in video data. For audio, wav2vec 2.0 (Baevski et al., 2020) provides robust speech-to-text and speech representation capabilities, enabling the study of conversational dynamics. Together, these models facilitate the efficient analysis of multimodal data. However, even the best model architecture needs diverse, high-quality datasets to learn from. A notable example of such a dataset is ImageNet (Russakovsky et al., 2015); in fact, the release of this dataset sparked the development of some of the most prominent computer vision models available today. Similarly, expanding publicly available datasets in developmental psychology contributes to accelerating progress in studying children’s everyday experiences.

Several publicly available datasets already exist. For example, the SAYCam dataset (Sullivan et al., 2021) provides audio-video recordings from three infants (6–32 months) who wore head-mounted cameras over 2 years, to capture naturalistic speech and behaviors. Similarly, the DAMI-P2C dataset (Chen et al., 2023) includes audio and video recordings of parent–child interactions during story reading, with annotations for body movements in a controlled environment. The MMDB dataset (Rehg et al., 2013) offers multimodal data (audio, video, physiological) of children (15–30 months) engaged in semi-structured play interactions, recorded in a lab. Another example is the UpStory dataset (Fraile et al., 2025), which features audio and video of primary school children (8 to 10 years) in dyadic storytelling interactions, also recorded in a lab setting. Finally, the BabyView dataset (Long et al., 2024) provides high-resolution, egocentric video of children aged 6 months to 3 years, recorded at home and in preschool environments, with annotations for speech transcription and pose estimation. Although these datasets vary in age, setting, and target behaviors, they collectively highlight the need for more naturalistic, at-home datasets that can capture the full range of children’s daily activities. Such datasets are particularly important because models, like the ones cited above, are usually trained on data from adults and therefore often fail to also capture, for example, speech or movement patterns of children with the same accuracy.

To address this gap, we introduce the publicly available ChildLens dataset, which focuses on activity annotations for children aged 3 to 5 years. The dataset consists of 108.58 h of video and audio recordings collected from 62 children in their home environment through a 140° wide-lens camera, integrated in a child-friendly vest (see also Zahra et al., 2024). It includes detailed annotations for five location classes and 14 activity classes, which are further categorized according to whether the child is interacting alone or with others. Designed to support research in developmental psychology and computer vision, the ChildLens dataset offers a rich resource for advancing multimodal learning and studying the full spectrum of children’s daily activities. At the time of submission, 51% (around 55 h) of the dataset has been annotated. The annotation process is ongoing and will be continued until the full dataset is annotated.

## Dataset generation

This section outlines the steps taken to create the ChildLens dataset. We provide detailed information on the video collection process, the labeling strategy employed, and the generation of activity labels.

### Step 1: Collection of egocentric videos

The ChildLens dataset consists of egocentric videos recorded by children aged 3 to 5 years over a period of 12 months. A total of 62 children from families living in a mid-sized city in Germany participated in the study. Each child was sampled only once at a specific age. However, within this single participation instance, there was a 1- to 2-week period during which the videos were recorded. While some children provided all material recorded across a single day, other children’s recordings were spread out over multiple days.

**Fig. 1:**
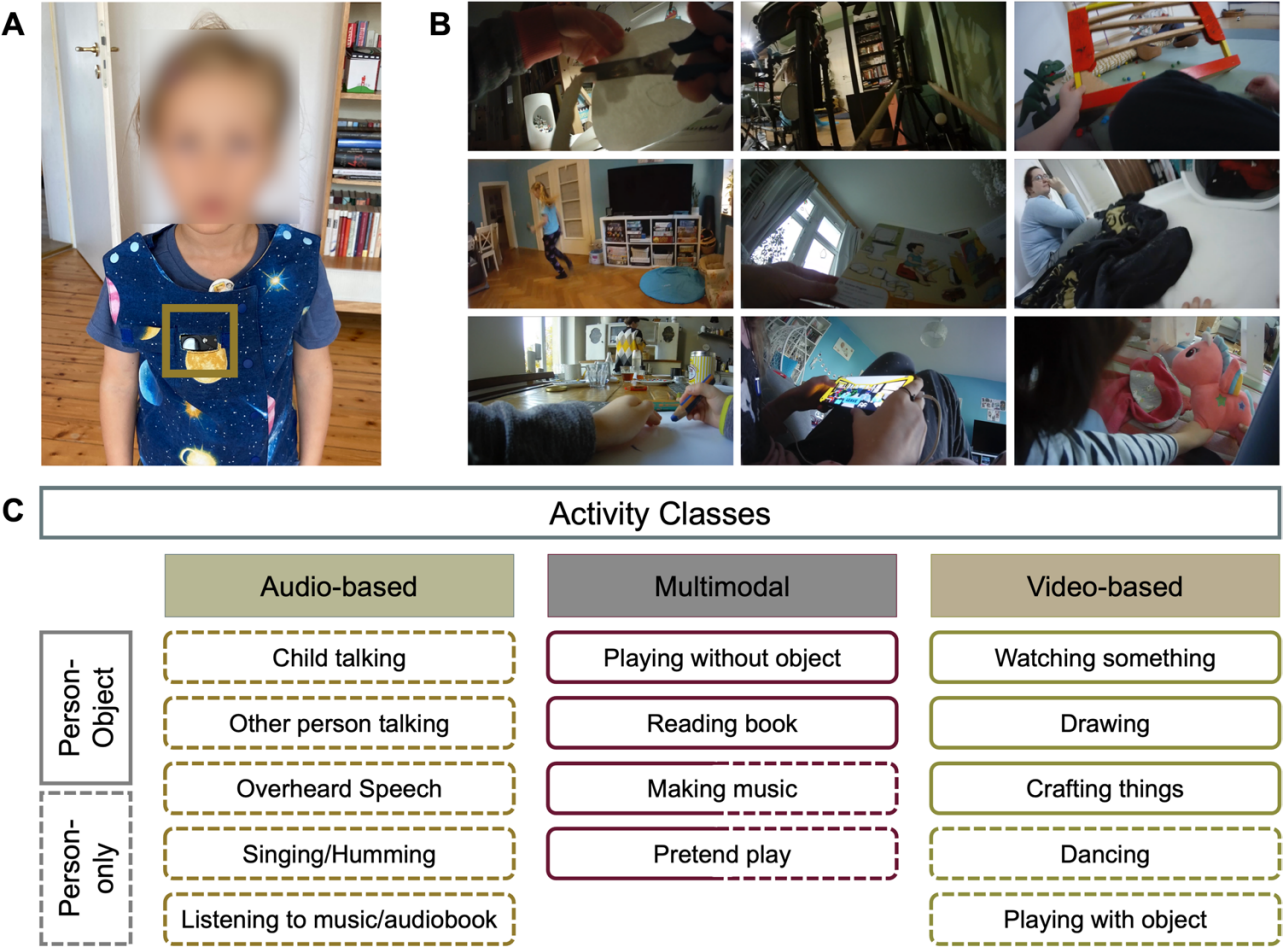
**A** Vest with the integrated camera worn by the children. **B** Collage of nine screenshots showing activities (top left to bottom right): crafting, dancing, drawing, making music, reading a book, watching something, playing with an object, playing without an object, and pretend play. **C** Activity classes in the ChildLens dataset

The videos were captured at home using a camera embedded in a vest worn by the children, as shown in Fig. 1. This setup allowed the children to move freely throughout their homes while recording their activities. The camera, a PatrolEyes Wi-Fi HD Infrared Police Body Camera, was equipped with a 140° wide-angle lens and captured the space in front of the child with a resolution of 1920 × 1080 p at 30 frames per second. The camera also recorded high-quality audio, allowing us to capture the child’s speech and other sounds in the environment.

In order to obtain decent coverage for the different activity classes we planned to annotate, we handed parents a short checklist of activities to record. The focus was on capturing everyday activities that children typically engage in. Parents were therefore asked to include the following elements in the recordings:

- Child spends time in different rooms and performs various activities in each room
- Child is invited to read a book together with an adult
- Child is invited to play with toys alone
- Child is invited to play with toys with someone else (adult or child)
- Child is invited to draw/craft something

### Step 2: Creation of labeling strategy

To create a comprehensive labeling strategy for the ChildLens dataset, we first defined a list of activities that children typically engage in. This list was inspired by previous research on activities that children are known to participate in (Ginsburg et al., 2007; Hofferth & Sandberg, 2001). From this, we derived a detailed catalog of activities that were likely to be captured in the videos and chose to make the activity classes more granular by distinguishing between activities like “making music” and “singing/humming” or “drawing” and “crafting things.”

After an initial review of the videos, we decided to add another class, “overheard speech,” to capture situations in which the child is not directly involved in a conversation but can hear it. We also added “pretend play” as a separate class to capture situations in which the child is engaged in imaginative play. This approach allowed us to capture the diversity of activities that children engage in and create a comprehensive dataset for activity analysis.

### Step 3: Manual labeling process

Before the actual annotation process, a setup meeting was held to introduce the annotators to the labeling strategy. To familiarize them with the task, the annotators were assigned 25 sample videos to practice and gain hands-on experience. These initial annotations were reviewed by the research team, and feedback was provided to refine the approach. A total of three feedback loops were conducted to ensure that the annotators followed the labeling strategy properly.

The videos were manually annotated by native German speakers who watched each video and labeled the activities present in the footage. Annotators marked the start and end points of each activity. For audio annotations, we implemented a two-second rule for all audio-based activity classes: if the break between two instances was 2 s or less, it was considered a single event; breaks longer than 2 s split the activity into separate instances.

## Dataset overview

### Activity classes

The ChildLens dataset includes 14 activity classes and five location classes. A brief description of each class can be found in Table 1. The location classes describe the current location of the child in the video and include *livingroom*, *playroom*, *bathroom*, *hallway*, and *other*. The activity classes are categorized based on the child’s interactions within the video and can be divided into *person-only* activities (e.g., “child talking,” “other person talking”) and *person-object* activities (e.g., “drawing,” “playing with object”). These activities are further categorized into *audio-based*, *visual-based*, and *multimodal* activities, as presented in Figure 1. Below is an overview of the different activity types:

- **Audio-based activities**: *child talking, other person talking, overheard speech, singing/humming, listening to music/audiobook*
- **Visual-based activities**: *watching something*, *drawing*, *crafting things*, *dancing*, *playing with object*
- **Multimodal activities**: *playing without object*, *reading book*, *making music*, *pretend play*

**Table 1:** Activity classes in the ChildLens dataset (first 12 child-centered, last 2 other-person-centered)

Activity class	Description
Child talking	Talking to themselves or to someone else
Singing/humming	Singing or humming a song or a melody
Listening to music/audiobook	Listening to music or an audiobook
Watching something	Watching a movie or video on either a screen or a device
Drawing	Drawing or coloring a picture
Crafting things	Engaged in a craft activity, such as making a bracelet
Dancing	Dancing to music or moving to a rhythm
Playing with object	Playing or interacting with an object, such as a toy or a ball
Playing without object	Playing without an object, such as playing hide-and-seek
Pretend play	Engaged in imaginative play, such as pretending to be a doctor
Reading a book	Reading a book or looking at pictures in a book
Making music	Playing a musical instrument or making music in another way
Other person talking	Another person is talking to the child
Overheard speech	Conversations the child can hear but is not directly involved in

### Statistics

**Fig. 2:**
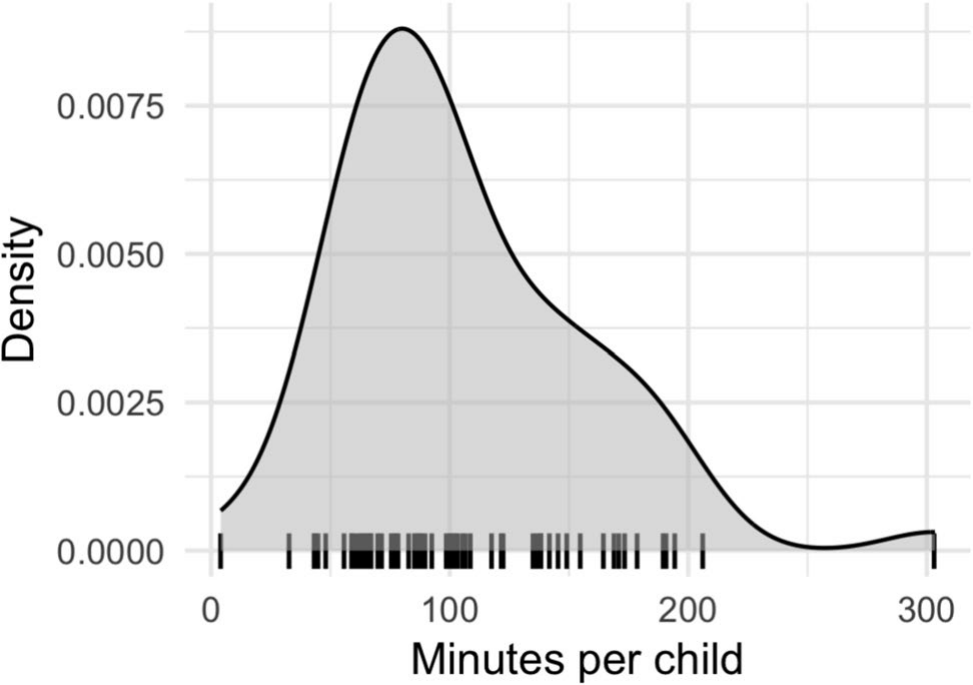
Distribution of video recording duration (in minutes) per child. Black vertical lines on the *x*-axis show the aggregated recording minutes for each child

The ChildLens dataset comprises 354 video files with a total of 108.58 h recorded by 62 children aged 3 to 5 years (*M* = 4.56, *SD* = 0.93), including 32 female and 30 male children. It contains 30.02 h from children aged 3, 41.91 h from children aged 4, and 36.66 h from children aged 5. The video duration per child varies between 4 and 303 min (*M* = 122.11, *SD* = 56.63). A detailed distribution of the video duration per child is shown in Fig. 2.

The dataset includes a varying number of instances with different total duration across the 14 activity classes. While annotations are still ongoing, the current annotations (51% annotated) include between 2.47 (making music) and 1,849.30 (playing with object) min per class. Depending on the activity class, the total duration for each activity class can consist of many shorter instances or fewer longer instances. For example, audio-based activities like “child talking” may last only a few seconds, while activities like “reading a book” can span several minutes. The total number of instances and summed duration for all activity classes is available in Table 4 in the Appendix.

### Exhaustive multi-label annotations

The dataset provides detailed annotations for each video file. These annotations specify the child’s current location within the video, the start and end times of each activity, the activity class, and whether the child is engaged alone or with somebody else. For every person involved in the activity, we capture age class and gender. If multiple activities occur simultaneously in a video, each activity is labeled individually. For example, if a segment shows a child “reading a book” while also “talking,” two separate annotations are created: one for “reading a book” and another for “child talking.” This exhaustive labeling strategy ensures that each activity is accurately represented in the dataset.

### Data availability

The ChildLens dataset will be made available to scientists for research purposes. It includes video and audio recordings, along with activity labels. Due to the sensitive nature of the data—recordings of children in their homes—access will be restricted.

Researchers can submit requests for access, which will be carefully reviewed to ensure proper handling and compliance with privacy standards. Please contact ra@eva.mpg.de to request access to the dataset. As noted above, the annotation process is still ongoing and the dataset will be updated regularly. A brief project overview, along with the latest dataset version, can be found on the ChildLens website: https://www.eva.mpg.de/comparative-cultural-psychology/technical-development/childlens/.

## Benchmark performance

In this section, we present the results of applying two model architectures to the currently annotated subset of the ChildLens dataset for two specific tasks: temporal activity localization using video data and voice type classification using audio data. For temporal activity localization, we used the Boundary-Matching Network (BMN) model (Lin et al., 2019), a state-of-the-art approach in this domain, which we trained from scratch on the unique activity classes in the ChildLens video data. For voice type classification, we applied and trained the Voice Type Classifier (VTC) (Lavechin et al., 2020), also state-of-the-art, using three different evaluation setups. Both models provide initial results and establish a benchmark for future research. The model architectures for both BMN and VTC are displayed in Figs. 5 and 6 in the Appendix, respectively.

All calculations were conducted on a Linux server equipped with two NVIDIA Quadro RTX 8000 GPUs, each with 48 GB of VRAM (CUDA version 12.5, driver version 555.42.06). The server had 48 CPU cores and 187 GB of RAM. The models were implemented in Python 3.8.18 for temporal activity localization and Python 3.8.19 for voice type classification using PyTorch (version.4.1+cu121 12.1), MMCV (version 2.2.0), and MMAction2 (version 1.2.0).

### Temporal activity localization

The Boundary-Matching Network generates action proposals by assigning confidence scores for predicted activity start and end boundaries. The architecture consists of two main components:

1. Proposal generation network: This network identifies candidate proposals. Each proposal is a set of start time and end time bounding a single potential activity bout within the video.
2. Proposal evaluation network: This network provides confidence scores for these proposals.

Crucially, the BMN does not classify these segments and therefore does not involve segment labeling.

#### Performance metrics

The model prioritizes proposals with high recall and high temporal overlap with ground truth. BMN performance is evaluated using average recall (AR), average number of proposals (AN), and area under the AR versus AN curve (AUC). AR is computed at various Intersection over Union (IoU) thresholds and for different AN as AR@AN, where AN ranges from 1 to 100. AR@100 reflects recall performance when the model is allowed to generate a maximum of 100 proposals per video.

The AUC metric quantifies the trade-off between the recall achieved and number of generated proposals. Specifically, the AUC is calculated by integrating the AR across the entire range of AN. This is conceptually similar to calculating the area under the precision–recall curve (AUC-PR) in classification, except that for the BMN the trade-off is between recall (how many ground truth activities were found) and the cost (how many total proposals were generated to find them).

Consider a 5-min video that contains two ground truth target activities: A (0:30–1:00) and B (4:30–5:00):

- Scenario 1 (High AUC): A model achieves 100% Recall (AR=1.0) by generating only two proposals (AN=2). Because the model achieves maximum recall with minimal proposals, its AUC score will be very high.
- Scenario 2 (Lower AUC): A model only achieves 50% Recall (AR=0.5) with its best 10 proposals (AN=10). It only reaches 100% Recall when forced to generate 50 proposals (AN=50). The need to generate many more proposals to get high recall penalizes the overall AUC score, even if the AR@100 remains the same. The AUC captures this inefficiency across the AN range.

On the ActivityNet-1.3 test set (Heilbron et al., 2015), BMN demonstrates effective activity localization with an AR@100 of 72.46 and an AUC of 64.47. A subsequent reimplementation of the model achieved slightly improved results (Xin, 2020), as displayed in Table 2.

**Table 2:** BMN performance on ActivityNet-1.3 and ChildLens datasets, showing average recall (AR) at different numbers of generated proposals (AR@1 to AR@100) and area under the curve (AUC)

Dataset	AR@1	AR@5	AR@10	AR@100	AUC
ActivityNet-1.3	33.6	49.9	57.1	75.5	67.7
ChildLens	**50.6**	**56.7**	**64.2**	**77.5**	**71.4**

#### Data preparation

The BMN implementation, including video preprocessing and model training, was conducted using the MMAction2 toolbox, an open-source toolbox for video understanding based on PyTorch (Contributors, 2020). Data preparation involved several key steps, such as raw frame extraction and the generation of both RGB and optical flow features for each video. Before training the model, we analyzed the distribution of activity instances across the classes for the annotated videos to assess the sufficiency of the data for both training and testing (see Table 4 in the Appendix).

Our analysis highlighted a significant class imbalance in the dataset, both in terms of instance count and the total duration of recordings. Given the primary goal of establishing initial benchmark results, no data augmentation methods were employed to mitigate this imbalance. Instead, we focused on the more frequent activity classes, which had the longest duration: “Playing with Object” (30.82 h of recording), “Reading a Book” (7.45 h of recording) and “Drawing” (6.1 h of recording).

For feature extraction and model training optimization, the videos were divided into clips of 4,000 frames (each clip corresponds to approximately 2 min 13 s). We then split these annotated clips into training, validation, and test subsets, using an 80-10-10 split. The training set was used for model optimization, the validation set guided hyperparameter tuning and overfitting prevention, and the test set was reserved for evaluating the model’s generalization ability on unseen data.

#### Implementation details

The BMN model was trained from scratch on the ChildLens dataset to predict the start and end boundaries of activity classes in the videos. The Adam optimizer was used with a learning rate of 0.001 and a batch size of 4. To avoid overfitting, early stopping based on validation loss was applied during training.

#### Evaluation

**Fig. 3:**
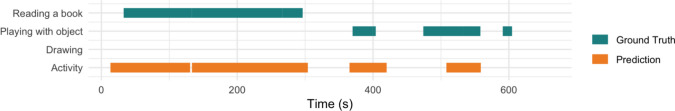
BMN predictions compared to ground truth annotations for a 605-s video snippet. The original video was split into five equally sized temporal snippets. Model predictions are in orange and ground truth annotations in green

The performance of the BMN model on the ChildLens dataset compared to its evaluation on ActivityNet-1.3 is summarized in Table 2, with AUC and AR reported for different numbers of generated proposals for both datasets. Figure 3 visualizes how model action localizations compare to the ground truth annotations. Across all proposal settings (AR@1 to AR@100), the BMN model achieves higher performance on ChildLens than ActivityNet. This result is expected, as the smaller number of activity classes (*n* = 3 vs. *n* = 200 for ActivityNet) and the shorter average video duration of the ChildLens dataset inherently make the task of temporal boundary localization less complex than on the ActivityNet-1.3 dataset.

However, this performance is considered promising due to the robustly high performance and demonstrates that the boundaries are reliably precise and consistently learnable by the BMN model. This is especially evident when evaluating at a lower number of proposals, which is more meaningful in this context. Even when limited to five proposals per video, the model achieves a strong AR@5 of 56.7 on ChildLens. This result underscores the quality and consistency of our annotations and confirms that the dataset provides a solid, well-balanced basis for training and evaluating temporal action proposal models on naturalistic egocentric videos from children.

### Voice type classification

Voice type classification is the task of identifying audio utterances and assigning them to predefined classes. In line with previous work, we focus on the following classes: Key Child (KCHI), Other Child (CHI), Male Speech (MAL), Female Speech (FEM), and Speech (SPEECH), as defined by the Voice Type Classifiers (VTC) output classes (Lavechin et al., 2020). Its architecture is composed of a SincNet layer, two bidirectional long short-term memory layers (LSTMs), and three feed-forward layers. The model takes a 2-s audio chunk as input and outputs a score between 0 and 1 for each class. The VTC was originally trained on the BabyTrain dataset, which includes 260 h of child-centered audio in multiple languages from children mostly aged 0 to 3 years.

The model architecture utilizes the open-source pyannote library for speaker diarization (Bredin, 2023; Plaquet & Bredin, 2023), which provides pretrained models and pipelines for various audio processing tasks. By adjusting the model architecture and training process using pyannote, we evaluated the ChildLens dataset’s quality through three distinct VTC training setups: In the first setup, we applied the pretrained VTC model directly to the ChildLens dataset. In the second setup, we fine-tuned the VTC model on the ChildLens data. In the last setup, we trained the VTC model from scratch using only the ChildLens dataset. These setups test the dataset’s annotation consistency and stand-alone value, with performance measured by the ${F}_{1}$-measure, a VTC-specific parameter (Bredin, 2017), which combines precision and recall.

#### Data preparation

We used the following mapping strategy to align our audio-based activity classes with VTC’s output classes. Minutes in parentheses indicate the total duration of annotated audio for each class.

- Child talking & Singing/humming → **Key Child** (963.44 min)
- Other person talking:
  - If age = "Child" → **Other Child** (53.55 min)
  - If age = "Adult" & gender = "Female" → **Female Speech** (492.96 min)
  - If age = "Adult" & gender = "Male" → **Male Speech** (223.41 min)
- Overheard speech, Child talking, Singing/humming, Other person talking → **Speech** (2158.24 min)

“Listening to music/audiobook” was excluded, as it is often background audio, lacks speaker age/gender details, and includes music irrelevant to VTC. “Overheard speech” refers to speech not directed at the key child and was mapped to SPEECH because no age/gender information was collected in the annotation process. This may underestimate VTC performance for cases when the VTC correctly predicts a woman speaking as FEM but is labeled as SPEECH. We retain this mapping, as re-annotation would be time-consuming. Moreover, the class is conceptually incompatible with the VTC output classes: it captures the recipient of speech rather than the speaker type. It is intended for more advanced models (for instance NLP architectures) that can infer not just who is talking, but also to whom, based on semantic content.

The ChildLens dataset was split into training, validation, and test sets, with 80% of the data used for training, 10% for validation, and 10% for testing. The training set was used to optimize the model parameters, the validation set guided hyperparameter tuning and overfitting prevention, and the test set was reserved for evaluating the model’s generalization ability on unseen data. To ensure speaker independence, the split was performed by child, resulting in audio data from 38 children in the training set and 10 each in the validation and test sets. This approach also maintained the approximate 80-10-10 distribution for each individual voice class.

#### Implementation details

For the three VTC setups, we used the original PyanNet architecture from pyannote.audio, which combines a SincNet frontend with a three-layer bidirectional LSTM (hidden size 128) and two fully connected layers (hidden size 128 each). We trained both $VT{C}_{ft}$ and $VT{C}_{cl}$ for 200 epochs. The only modification we made was to the offset parameter, which defines how long a class must remain inactive before it is considered “off.” We increased this value from 0.1 to 2.0 to align with our two-second annotation rule.

#### Evaluation

Table 3 shows the ${F}_{1}$-measure for three VTC setups on the ChildLens dataset—the original model $VT{C}_{og}$, the fine-tuned model $VT{C}_{ft}$, and the model trained from scratch $VT{C}_{cl}$—compared to the benchmark dataset. $VT{C}_{og}$ scores 45.30, slightly below the benchmark, with highest performance on KCHI (67.10) and lowest on CHI (5.80). Both $VT{C}_{ft}$ and $VT{C}_{cl}$ achieve noticeably higher scores (51.60 and 52.30, respectively), indicating significant improvement compared to the $VT{C}_{og}$ ChildLens performance. While the higher performance of $VT{C}_{ft}$ and $VT{C}_{cl}$ might be expected, the successful adaptation across all training setups provides solid evidence for the high quality of the ChildLens annotations: achieving a satisfactory baseline score, which demonstrates that our annotations are structurally coherent and recognizable by an external audio model. Furthermore, the performance jump observed when training data contained ChildLens data $VT{C}_{ft}$ and $VT{C}_{cl}$ confirms the necessary internal consistency for the labels to be robustly learned for the classification task.

**Table 3:** Comparison of Voice Type Classifier (VTC) performance on the ACLEW-Random dataset and the three setups utilizing the ChildLens dataset. The evaluation metrics are reported for the original VTC model (VTC-OG), the VTC fine-tuned on ChildLens (VTC-FT), and the VTC trained from scratch on ChildLens (VTC-CL). The table reports the *F*_1_-measure per class and the average *F*_1_-measure (AVG)

Dataset	Model	KCHI	CHI	MAL	FEM	SPEECH	AVG
ACLEW-Random	VTC-OG	68.7	**33.2**	**42.9**	**63.4**	78.4	**57.3**
ChildLens	VTC-OG	67.1	5.8	22.2	44.3	87.2	45.3
ChildLens	VTC-FT	76.3	5.5	35.3	52.5	88.4	51.6
ChildLens	VTC-CL	**76.6**	6.1	36.4	53.3	**88.9**	52.3

Looking at the results in more detail, the low CHI scores are primarily due to frequent misclassifications as KCHI. This likely results from the vocal similarity between the key child and other children in the ChildLens dataset. In many cases, distinguishing between them may rely more on acoustic cues like proximity to the microphone than on vocal characteristics. Moreover, CHI is by far the smallest voice type class, with only 53.55 min of annotated data in total, which makes it especially difficult for the model to learn robust representations. Consequently, our ability to evaluate how well the model generalizes to unseen examples of this class is also limited, as the test set contains only 3.57 min of “other child” speech.

**Fig. 4:**
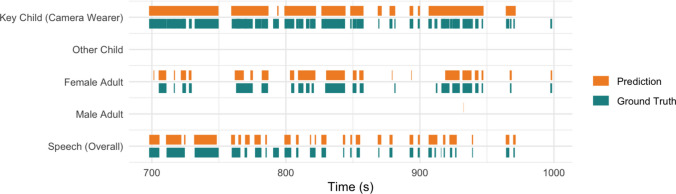
$VT{C}_{cl}$ predictions compared to ground truth annotations for a 300-s video segment. The plot displays the temporal occurrences for the five voice types (*y*-axis) and concurrence between the model’s predictions (orange) and the ground truth annotations (green)

**Fig. 5:**
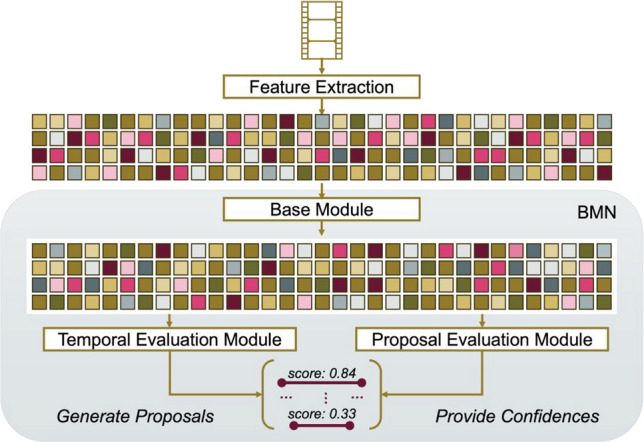
Boundary Matching Network Architecture (Lin et al., 2019)

**Fig. 6:**
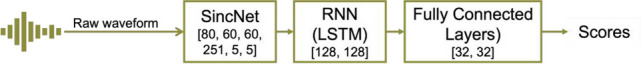
Voice Type Classifier Architecture (Lavechin et al., 2020)

In contrast, the original VTC dataset includes 327 min of CHI data for training, and contains audio from infants and toddlers, where the key child often sounded younger or was babbling. This makes it easier to tell them apart from other speakers, which likely helped the model perform better on this class. Importantly, these scores are expected to improve as more annotated data become available; as mentioned earlier, the annotation process is still ongoing. Figure 4 visualizes how model predictions compare to the ground truth annotations for the $VT{C}_{cl}$ model.

## General discussion

We present the ChildLens dataset, an egocentric video-audio dataset that documents naturalistic everyday experiences in preschool children. This dataset is particularly distinctive due to its diversity in terms of the number of children it includes and the variety of activity labels it covers. The ChildLens dataset provides comprehensive audio and video annotations for a broad spectrum of key activities, including multimodal social interactions. These annotations support the training and evaluation of models for the automatic analysis of children’s activities and therefore allow for scaling up data collection.

In comparison to other freely available datasets, the ChildLens dataset stands out due to its broad age span and diverse set of activity labels. Most existing datasets focus on toddlers, are limited to dyadic interactions, or were recorded in lab settings, with all of them lacking a comprehensive range of activity labels. Furthermore, most of these datasets capture either only audio or video. In contrast, ChildLens includes naturalistic recordings from children’s home environments, collected over an extended period, and features a wide variety of activity types. Particularly noteworthy is the activity class Overheard Speech, which captures speech that children can hear but are not directly involved in. This class is important for studying the impact of overheard speech on children’s cognitive development—an area that has largely relied on labor-intensive manual annotations due to the difficulty of automatically distinguishing between child-available and child-directed speech (Bergelson et al., 2023). Existing models, like the classifier developed by Bang et al. (2023), could be enhanced by incorporating visual features—such as eye contact or the use of gestures—in addition to audio inputs. The ChildLens dataset also captures whether children are engaged in activities alone or with others and provides basic demographic information—age and gender—about all individuals involved.

The usefulness of the ChildLens dataset is demonstrated by its successful application to well-established models. For example, the Voice Type Classifier for audio classification achieves performance comparable to the previous benchmark dataset, while the BMN produces robust results for activity localization, consistent with its performance on widely used datasets such as ActivityNet. One way of using the ChildLens dataset to advance methodological development is through multi-method approaches. For example, activity localization could be further enhanced by incorporating object identification, allowing for better tracking of the objects children interact with during daily routines. Such an approach has been used in adult-focused studies (Kazakos et al., 2021). Research by Bambach et al. (2015) also emphasizes the importance of hand detection in egocentric video for activity recognition. Their use of convolutional neural networks (CNNs) for hand segmentation demonstrates how such techniques can help differentiate between activities. To apply a similar approach to the ChildLens dataset, we would first need to run a pretrained hand detection model on the dataset. Afterwards, we would train a CNN to classify frames containing hands into activity classes, following the method described by Bambach et al. (2015). If the performance of the hand detection performance is insufficient, additional hand annotations would be required to improve the model’s accuracy. A second use case of the ChildLens dataset is for activity classification. The BMN proposes start and end points of activities, but does not classify these activities. The annotations included in the dataset do provide labels for each activity and can thus be used to develop and train model architectures that classify activities typical for children. Such an approach would be particularly relevant, as most existing datasets and models are focused on adult activity recognition and temporal localization. These typically include activities from broad domains such as household tasks (e.g., making a sandwich, vacuuming; Heilbron et al., 2015), sports activities (e.g., golf swing, high jump; Idrees et al., 2017), or surveillance-related actions (e.g., opening car door, texting on the phone; Lara & Labrador, 2013).

The integration of visual and auditory data in the ChildLens dataset enables a more detailed and comprehensive understanding of children’s daily experiences. Complex activities such as pretend play and reading a book, which require both audio and video for accurate detection, exemplify the strength of this multimodal approach. While previous studies, such as those analyzing disfluencies in children’s speech during computer game play (Yildirim & Narayanan, 2009), have demonstrated that combining visual and auditory information can improve performance, few studies have explored this in the context of children’s naturalistic activities. With ChildLens, the combination of naturalistic data and multimodal analysis creates new opportunities for in-depth insights into children’s cognitive, emotional, and social development, particularly for activities best captured through both modalities.

Despite its strengths, the ChildLens dataset also has its limitations. First, there is class imbalance, especially in underrepresented activity classes, which could affect model training and evaluation. More frequent activities, such as “playing with object” (1,849 min) and “reading a book” (447 min), dominate the dataset, whereas less common activities like “dancing” (9 min) and “making music” (2 min) are scarcely represented. Similarly, activities like “playing without object” (48 min) and “watching something” (28 min) appear less frequently. This imbalance may lead to skewed model performance, making it harder to accurately classify rare activities. Possible solutions to this challenge could involve merging rare activity classes into broader categories or excluding them from model training, though these approaches may reduce the dataset’s diversity. Other methods, such as resampling or augmentation, could help balance the dataset and improve model performance (Alani et al., 2020; Spelmen & Porkodi, 2018). Second, there is sampling bias. Since the recordings are largely influenced by parental decisions about when and how often activities are captured, certain activities or settings may be overrepresented or underrepresented on the basis of these preferences. Furthermore, the dataset focuses primarily on families from a mid-sized German city, limiting its geographical and cultural diversity. Expanding the dataset to include a broader range of families from different regions and cultures would enhance its generalizability and applicability to various research contexts.

The study of children’s everyday experiences is crucial for understanding their cognitive, emotional, and social development. These daily interactions provide important insights into how children learn, grow, and engage with their environment. The ChildLens dataset makes a valuable contribution to this field by offering a rich multimodal resource that captures children’s experiences in naturalistic settings. With its comprehensive annotations and potential to automate the analysis of children’s activities, the dataset enables researchers to develop, and apply automized processing algorithms that help to scale up the study of child development. By virtue of being an openly accessible resource, the ChildLens dataset creates new opportunities for understanding the complexities of early childhood development and provides a foundation for future research in this area.

## Data Availability

The data are available at 10.17617/4.fe. Access must be requested via email to ra@eva.mpg.de and will be granted following a review process. An overview of the dataset can be found at https://www.eva.mpg.de/comparative-cultural-psychology/technical-development/childlens/. Access to this repository is managed by the Max Planck Institute for Evolutionary Anthropology’s research team, rather than an individual researcher. This institutional oversight ensures that the data will be maintained and available for future use, regardless of any personnel changes.

## Appendix

### Activity class and location statistics

**Table 4:** Number of video instances and the total duration in minutes for the number of currently annotated videos (n=192)

Category	Activity Class	Instance Count	Total Duration (min)
Audio	Child talking	12093	839.04
	Other person talking	10223	771.25
	Overheard Speech	3285	424.88
	Listening to music/audiobook	96	322.88
	Singing/Humming	361	104.01
Video	Drawing	83	366.30
	Crafting things	28	107
	Watching something	5	27.80
	Dancing	21	8.75
Multimodal	Playing with object	498	1849.30
	Reading a book	104	447.10
	Pretend play	68	182.26
	Playing without object	38	47.94
	Making music	3	2.47
Location	Livingroom	112	1590.99
	Playroom	71	998.73
	Other Room	49	559.88
	Hallway	18	42.47
	Bathroom	2	2.25

## Code availability

The code used to generate the benchmark performances is available on our GitHub page (https://github.com/neleSuffo/ChildLens).
